# Identification of R2R3-MYB Transcription Factor Family Based on *Amaranthus tricolor* Genome and *AtrMYB72* Promoting Betalain Biosynthesis by Directly Activating *AtrCYP76AD1* Expression

**DOI:** 10.3390/plants14030324

**Published:** 2025-01-22

**Authors:** Yuwei Xue, Kexuan Li, Wenli Feng, Zhongxiong Lai, Shengcai Liu

**Affiliations:** 1 Institute of Horticultural Biotechnology, Fujian Agriculture and Forestry University, Fuzhou 350002, China; xueyuwei_x@163.com (Y.X.); pahwhy@163.com (K.L.); fwl9007@163.com (W.F.); laizx01@163.com (Z.L.); 2 Key Laboratory of Ministry of Education for Genetics, Breeding and Multiple Utilization of Crops, Fujian Agriculture and Forestry University, Fuzhou 350002, China

**Keywords:** *Amaranthus tricolor* L., R2R3-MYB family, *AtrMYB72*, betalain biosynthesis

## Abstract

MYB (myeloblastosis) is one of the most abundant transcription factors in plants which regulates various biological processes. The molecular characteristics and function of R2R3-MYB transcription factors in amaranth remain unclear. In this study, 73 R2R3-MYB members were identified from the amaranth genome database and we further analyzed their chromosome position, conserved motifs, physiological and biochemical features, collinearity relationships, gene structure, phylogeny and *cis*-acting element. Based on the phylogenetic and expression pattern analysis, 14 candidate *R2R3-MYB* genes might be involved in the betalain synthesis. Amongst the 14 candidate *R2R3-MYB* genes, the expression level of *AtrMYB72* was higher in ‘Suxian No.1’ than ‘Suxian No.2’, and also higher in the red section than in the green section of the same leaf in *Amaranthus*. The overexpression vector pCambia1301-*AtrMYB72*-GUS and VIGS (virus-induced gene silencing) vector pTRV2- *AtrMYB72* were transferred into leaves of ‘Suxian No.1’ via an *Agrobacterium*-mediated method. The results showed that *AtrMYB72* overexpression could promote betalain synthesis. A yeast one-hybrid assay and dual luciferase reporter gene assay demonstrated that *AtrMYB72* could bind to the *AtrCYP76AD1* promoter to promote betalain synthesis. These results indicated that *AtrMYB72* promoted betalain biosynthesis in amaranth by activating the *AtrCYP76AD1* transcription. Our results could provide new insights into the betalain biosynthesis in amaranth.

## 1. Introduction

*Amaranthus tricolor* L. is an herbaceous plant of the family Amaranthaceae, which is widely distributed in warm and tropical regions worldwide [1]. The amaranth can adapt to various climatic conditions with no major disease problems. And the amaranth plant also has important nutritional and medicinal properties [2]. Its leaves are utilized as vegetables containing betalain, flavonoids, alkaloids and other elements, exhibiting anti-oxidative and radical-scavenging properties [3,4,5,6]. *Amaranthus tricolor* becomes a promising crop for the future. Moreover, *Amaranthus* plants are substituted for beet as a source for extracting natural betalain [7,8].

Betalain, a water-soluble nitrogen-containing pigment, generally exists in vacuoles [9,10]. It is divided into betacyanin and betaxanthin [11]. Betalain has important antioxidant, antiviral and anti-inflammatory effects [12,13,14,15]. It can serve as an important osmotic substance and non-enzymatic antioxidant through scavenging excess reactive oxygen species (ROS) to resist (a)biotic stress such as high temperature, drought and other adverse environments, maintaining the normal physiological activities in plants [16,17].

Betalain is synthesized from L-tyrosine, which is the precursor of betalain biosynthesis [18]. Initially, L-tyrosine is hydroxylated to L-DOPA by Cytochrome P450 enzyme CYP76AD [19]. Then, L-DOPA is catalyzed to an open cyclo-DOPA by 4,5-dopa dioxygenase (DODA) and spontaneously forms betalamic acid [20]. The betalamic acid can spontaneously combine with amino acids or amines to produce betaxanthin. Meanwhile, cyclo-DOPA is formed by the cytochrome P450 enzyme CYP76AD1 oxidized dopamine, and cyclo-DOPA spontaneously condenses to form beta-glucoside ligand [21,22,23]. Finally, betacyanin is generated under the action of beta-glucoside ligand-5-O-glucosyl transferase (B5GT) [24]. At the same time, cyclo-DOPA is catalyzed to cyclo-DOPA-glucoside by cyclo-DOPA 5-O-glucosyltransferase (cDOPA5GT), then, cyclo-DOPA-glucoside spontaneously condenses with betalamic acid to form betacyanin [25,26].

Transcription factors, such as MYB [27,28], WRKY [29,30], SPL [31] and bHLH [32], are involved in the betalain biosynthesis. WRKY transcription factor *HmoWRKY40* could bind and activate the promoter of *HmoCYP76AD1* to participate in betalain biosynthesis [30]. *AtrWRKY42-2* transcription factor could interact with *AtrCYP76AD1* to regulate betalain biosynthesis in *Amaranthus tricolor* [33]. bHLH transcription factor *HubHLH159* is involved in the regulation of betalain biosynthesis, which could promote the synthesis through activating the gene expression of *HuADH1*, *HuCYP76AD1-1* and *HuDODA1* [32]. *HuSPL12* could interact with *HuMYB1*, *HuMYB132* or *HuWRKY42* TFs responsible for betalain biosynthesis [31].

As one of the largest transcription factor families in higher plants, MYB transcription factor is divided into four subfamilies (1R-, 3R-, 4R- and R2R3-MYB, respectively) based on incomplete MYB domain repeats (R) [34,35]. And the MYB transcription factor consists of three helix-turn-helix structures (HTH) of approximately 53 amino acids per conserved repeat. R2R3-MYB transcription factors are the largest number in the MYB family in the plant [36,37]. R2R3-MYB proteins can regulate the transcription of downstream gene through interaction with *cis*-acting elements in the promoter region of target genes, which are involved in the regulation of (a)biotic stress, plant growth and development, primary metabolism and secondary metabolism [38,39,40,41,42]. Betalain is a kind of secondary metabolite in plants, and its synthesis is also regulated by R2R3-MYB transcription factors [43]. In beet, *BvMYB1* activated the promoter region of the cytochrome P450 gene *BvCYP76AD1* and 4,5-dopa dioxygenase gene *BvDODA1* to regulate betalain biosynthesis [28]. In pitaya, *HuMYB1* [44] and *HuMYB9* [45] could reduce *HuADH1*, *HuCYP76AD1-1* and *HuDODA1* transcriptional activities, so they inhibited the synthesis of betalain biosynthesis in the mature pitaya pulp. These research suggest that R2R3-MYB regulating betalain synthesis is a very complex process.

The *R2R3-MYB* genes of *AmMYB1* (GenBank: KU557504.1) [46] and *AmMYB2* (GenBank: KY814751.1) [47] were cloned in amaranth, which were perhaps involved in betalain biosynthesis, but the experimental verification is lacking. It is still unknown if the mechanism of betalain biosynthesis was regulated by MYB transcription factor in amaranth. In this study, we identified R2R3-MYB transcription factor based on amaranth genome, and used qRT-PCR to screen candidate R2R3-MYB members which were involved in betalain biosynthesis. Finally, we analyzed the regulation mechanism of *AtrMYB72* using transient transformation assay, yeast one-hybridization assay and dual-luciferase transient expression assay. The results could provide new insights into the betalain biosynthesis in amaranth.

## 2. Results

### 2.1. Identification and Physical Parameters of R2R3-MYB Transcription Factor in Amaranth

**Figure 1:**
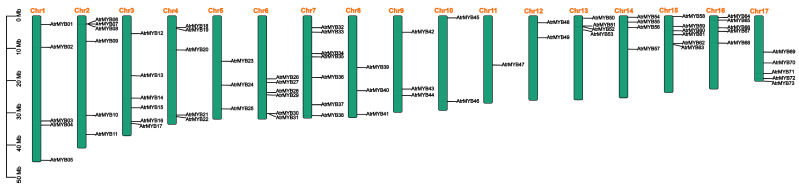
Distribution of *Amaranthus tricolor R2R3-MYB* (*AtrMYB*) genes among 17 chromosomes. Gene positions and the size of each chromosome can be estimated using the scale on the right of the figure; the scale indicates 10 megabases (Mb).

A total of 211 candidate deduced amino acid sequences containing MYB or MYB-like repeats were obtained from the amaranth genome database. The MYB domains were subsequently analyzed by Pfam and SMART. In total, 73 R2R3-MYB proteins were obtained. Genome chromosomal location analyses showed that the 73 *R2R3-MYB* genes were distributed throughout 17 chromosomes (Chrs) in the amaranth genome and renamed as *AtrMYB01* to *AtrMYB73* according to their chromosome location (shown in Figure 1). The largest number of MYB genes (seven) was mapped on Chr 7.

Furthermore, the protein sequence length ranged from 125 (*AtrMYB49*) to 1017 (*AtrMYB68*) amino acids, the computed molecular weights of these R2R3-MYB proteins ranged from 17.48 (*AtrMYB49*) to 112.94 kDa (*AtrMYB68*). The theoretical pI of R2R3-MYBs is between 4.80 (*AtrMYB61*, *AtrMYB72*) and 9.68 (*AtrMYB49*). The instability coefficient is between 38.72 (*AtrMYB42*) and 74.46 (*AtrMYB17*), and most AtrMYBs members (68.49%, 68/73) were unstable (instability index > 40). The subcellular localization of all R2R3-MYBs proteins was predicted in the nucleus.

### 2.2. Multiple Sequence Alignment

**Figure 2:**
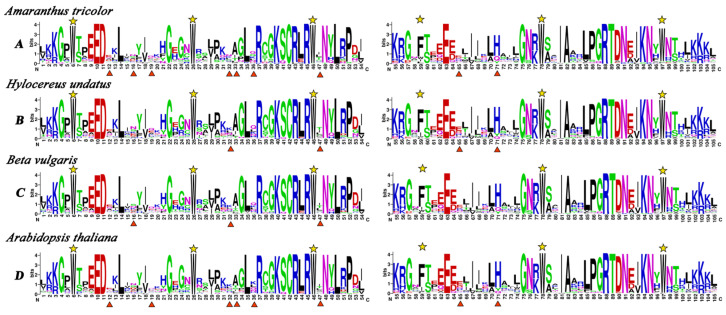
Comparison of DNA-binding domains of R2R3-MYB transcription factor in *Amaranthus tricolor*, *Hylocereus undatus*, *Beta vulgaris* and *Arabidopsis thaliana*. Sequence logos of the R2 and R3 repeats are based on conserved alignments from *Amaranthus tricolor* (**A**), *Hylocereus undatus* (**B**), *Beta vulgaris* (**C**) and *Arabidopsis thaliana* (**D**). The overall height of each stack indicates the conservation of the sequence at the position, whereas the height of letters within each stack represents the relative frequency of the corresponding amino acid. Highly conserved tryptophan (W) and phenylalanine (F) residues are indicated by yellow asterisks. The positions with different patterns between *Amaranthus tricolor*, *Hylocereus undatus*, *Beta vulgaris* and *Arabidopsis thaliana* are indicated by arrows. The positions with different patterns between *Amaranthus tricolor*, *Hylocereus undatus*, *Beta vulgaris* and *Arabidopsis thaliana* are indicated by red triangle.

To investigate the homologous domain sequence features, conservation and divergence of R2 and R3 repeats from amaranth (73), pitaya (105), beet (70) and *Arabidopsis thaliana* (126) were assessed using multiple alignment analyses (Figure 2). The results showed that tryptophan (W) residues in the R2 and R3 repeat sequences of *AtrMYBs* were highly conserved and evenly distributed, which were consistent with AtMYBs, BvMYBs and HuMYBs. In amaranth, the conserved motifs of the R2 protein domains are [W]-x(19)-[W]-x (19)-[W], and the conserved motif of the R3 protein domain is [F]-x(18)-[W]-x(18)-[W] (Figure 2A). Three conserved W residues in the R2 repeat sequence (position 6, 26 and 46), and the R3 repeat sequence had two conserved W residues (position 78, 97), and the W residue at the position 59 is replaced by the phenylalanine (F) residue (Figure 2A). The domain of R2R3 is found in *Arabidopsis thaliana*, beet and pitaya, indicating that the evolution of R2R3-MYB transcription factor is conserved among plants (Figure 2B–D). The conserved residue (position 32) in the R2 repeat sequence of amaranth is different from *Arabidopsis thaliana*, beet and pitaya. Furthermore, the conserved residue (position 36) of amaranth, pitaya and beet is different from *Arabidopsis thaliana*. Compared to *Arabidopsis thaliana*, the residue at the position of 12, 19, 32, 33, 47 and 36 in R2 repeat sequence, and at the position of 65 and 71 in R3 repeat sequence in amaranth is different. The residue at the position of 16, 32 and 47 in R2 repeat sequence is different between amaranth and beet. Meanwhile, the residue at the position of 32 and 47 in R2 repeat sequence, and 65 and 71 in R3 repeat sequence is different between amaranth and pitaya. The results indicated that the difference in the MYB domain between amaranth and *Arabidopsis thaliana* was greater than that between amaranth and pitaya or beet. The results suggest a closer relationship between amaranth and pitaya or beet, while also reflecting the species-specific differences.

### 2.3. Collinearity Analysis of the R2R3-MYB Gene Family

**Figure 3:**
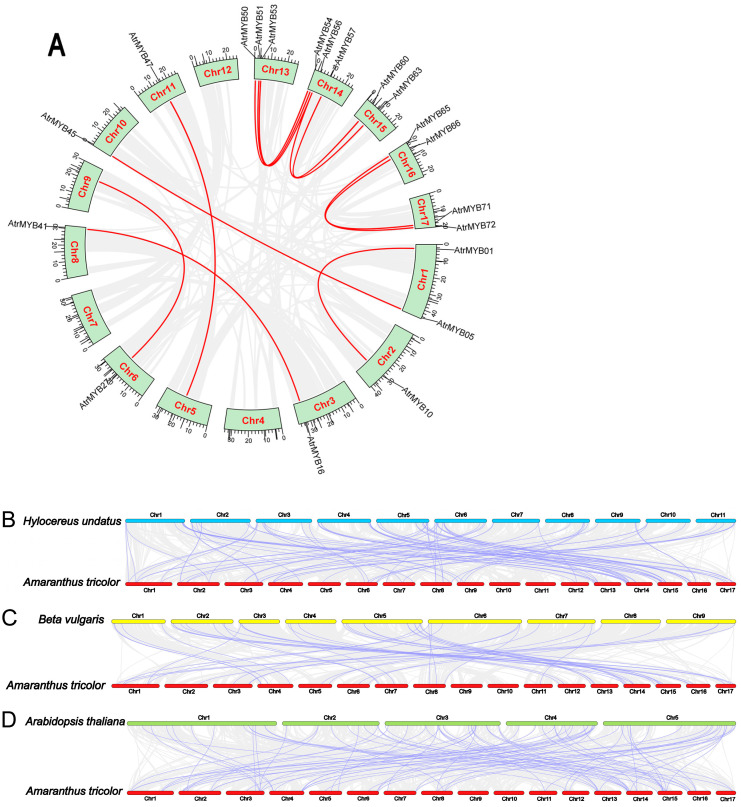
Collinearity analyses of *R2R3-MYB* genes. (**A**) Segmental duplication events of *R2R3-MYB* genes in amaranth. (**B**) Duplication events of *R2R3-MYB* genes between amaranth and pitaya. (**C**) Duplication events of *R2R3-MYB* genes between amaranth and beet. (**D**) Duplication events of *R2R3-MYB* genes between amaranth and *Arabidopsis thaliana*. Purple lines indicate duplication events of *R2R3-MYB* genes. Gray lines represent all synteny blocks in genomes.

Analysis of genome-wide replication is crucial for understanding the genesis, evolution and genome-wide expansion of organisms. To further understand the reasons behind gene replication events in *AtrMYBs*, we therefore examined the replication events of the *R2R3-MYB* gene family in amaranth. In this study, Chr 4, Chr 7 and Chr 12 were chromosomes without fragment repeats; 12 *AtrMYBs* with large fragment repeat segmental duplication events with 20 pairs. These findings suggested that the amplification and evolution of the *R2R3-MYB* genes in the amaranth genome may have been significantly influenced by the fragment replications; syntenic duplications mainly contribute to the expansion of *AtrMYB* genes (Figure 3A).

We conducted a synteny analysis to understand the evolution relationships of amaranth R2R3-MYB family members with other plants, including *Arabidopsis thaliana*, beet and pitaya. In total, 89 collinear *R2R3-MYB* genes pairs were identified between amaranth and pitaya (Figure 3B), 46 were obtained between amaranth and beet (Figure 3C) and there were 83 genes between amaranth and *Arabidopsis thaliana* (Figure 3D). Based on the analysis of collinearity, amaranth is more closely related to pitaya than to *Arabidopsis thaliana* and beet, evolutionarily closer to pitaya.

### 2.4. Phylogenetic Analysis and Structural Classification of R2R3-MYB Genes

**Figure 4:**
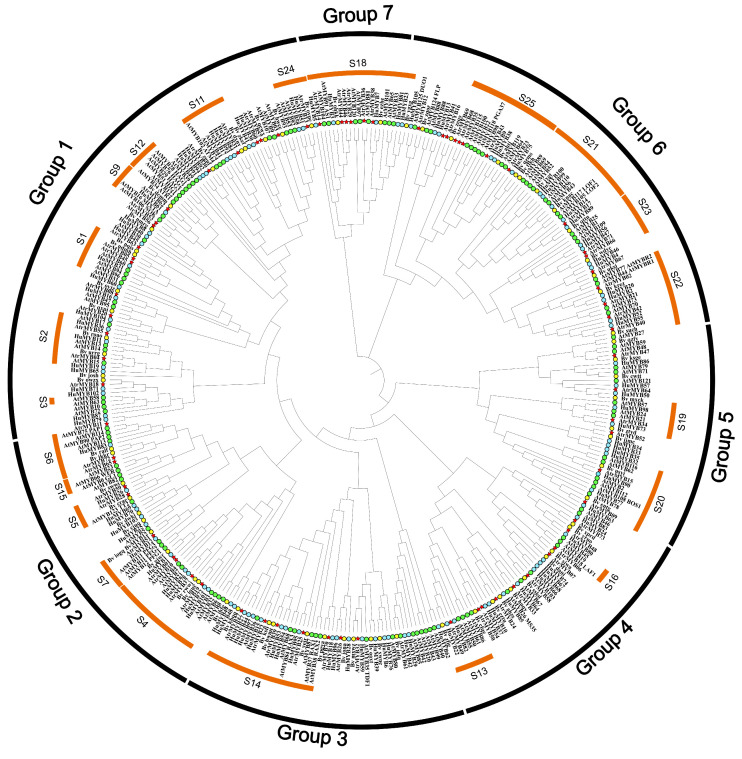
Phylogenetic relationships of R2R3-MYBs. *Arabidopsis thaliana*, amaranth, pitaya and beet R2R3-MYBs were used for the phylogenetic tree construction using the ML method. Red stars represent the R2R3-MYBs of amaranth, blue circles represent the R2R3-MYBs of pitaya, green circles symbolize the R2R3-MYBs of *Arabidopsis thaliana* and yellow circles represent the R2R3-MYBs of beet.

To understand the phylogenetic relationships of the AtrMYB proteins, an evolutionary tree of 73 AtrMYBs, 105 HuMYBs, 70 BvMYBs and 126 AtMYBs was used to construct a phylogenetic tree using the ML method (Figure 4). All *R2R3-MYB* genes were divided into 25 subgroups (designated as S1~S25, excluding S8, S17). AtrR2R3-MYBs were found in 18 of these subgroups, excluding S3, S12, S15, S16, consistent with beet and pitaya. Among them, S18 has the maximum number of family members with 6 AtrR2R3-MYBs.

**Figure 5:**
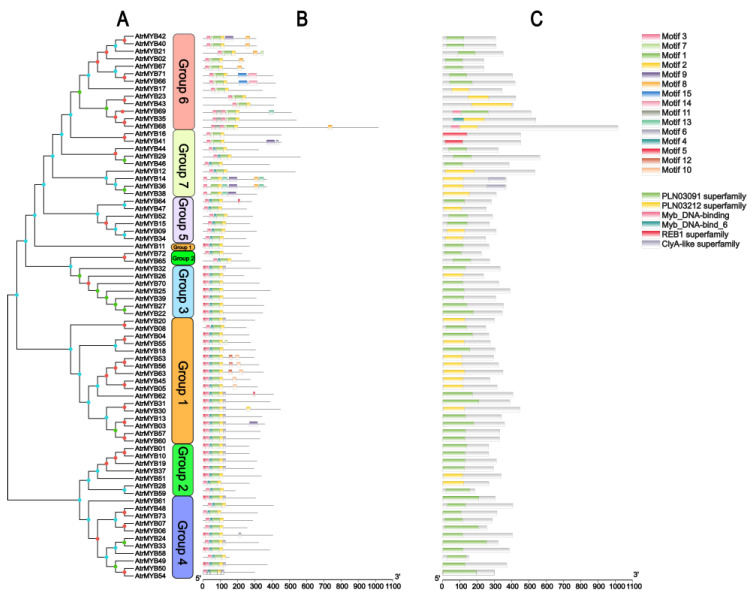
AtrR2R3-MYBs phylogenetic relationship (**A**), conserved motifs (**B**), and conserved domains (**C**). Orange circles indicate the bootstrap value range from 81 to 100 in the tree, green is from 60 to 80, and blue is from 0 to 59.

According to the phylogenetic tree topology, 73 AtrMYB proteins were classified into seven groups (designated as Group 1 to Group 7) (shown in Figure 5A). By detecting the motif compositions of AtrR2R3-MYB protein characteristic regions, we identified 15 conserved motifs of *R2R3-MYB* genes using the MEME online website (Figure 5B). The most conserved motifs were located in the N-terminus of R2R3-MYB proteins. The number of conservative motifs in each AtrR2R3-MYB ranged from 4 to 7. Motif 1, Motif 2 and Motif 3 were found in all ArtR2R3-MYB. The most closely related members exhibited similar motif compositions and conserved domains (Figure 5C), which indicated that the members in the same subgroup might perform similar functions.

### 2.5. Cis-Regulatory Elements Analysis of the R2R3-MYB Promotors in Amaranth

**Figure 6:**
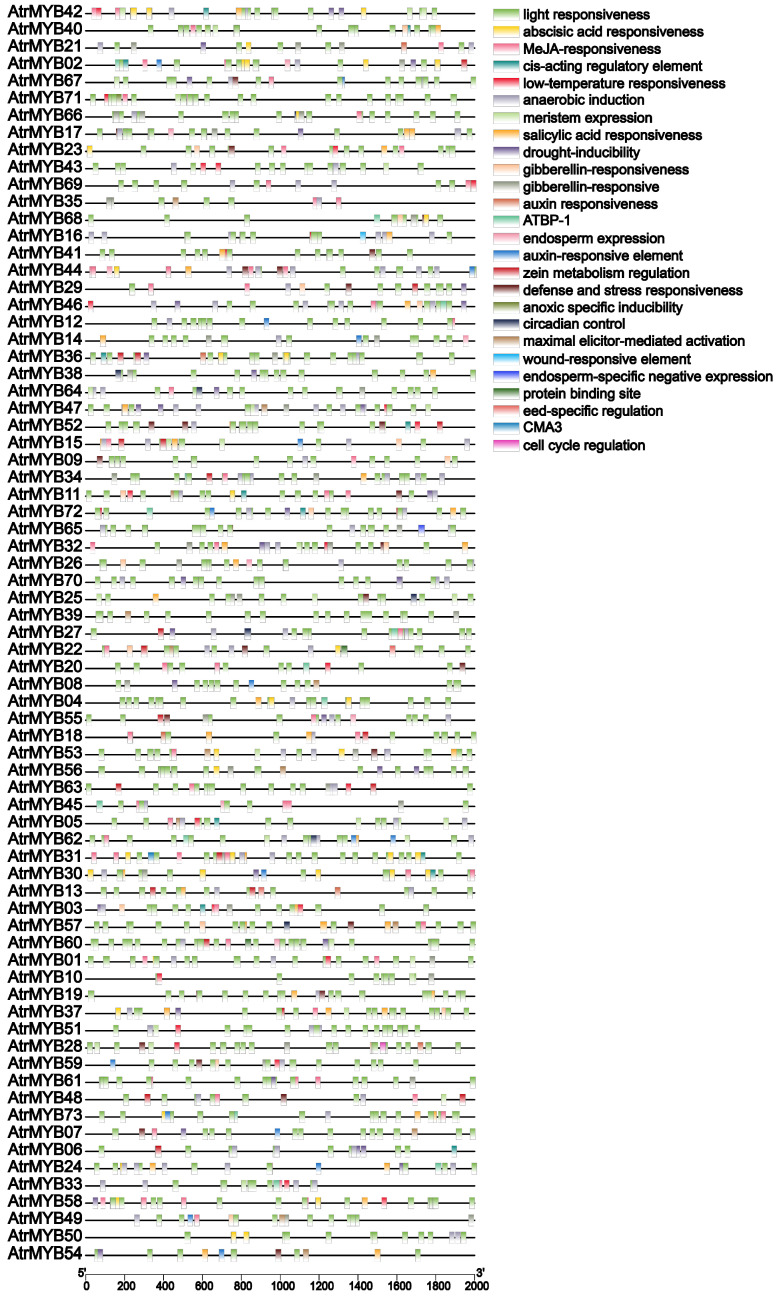
The regulatory element of R2R3-MYB gene promoters in amaranth.

To understand the potential regulatory mechanisms of AtrMYBs, *cis*-regulatory elements were predicted using PlantCARE. The prediction of *cis*-acting elements shows that the *cis*-acting elements were involved in the light-responsive, ABA-responsive, MeJA-responsive, auxin, gibberellin, salicylic acid, drought, low temperature, defense, wound responsiveness, organ-specific expression of seeds, endosperm, roots and meristem expression, anaerobic induction, circadian rhythm, cell cycle regulation, flavonoids biosynthesis and other regulatory elements (Figure 6). The analysis showed that *R2R3-MYB* genes are involved in environmental stress and phytohormones, making it possible to further study gene functions.

### 2.6. Expression Pattern of AtrMYB Genes

**Figure 7:**
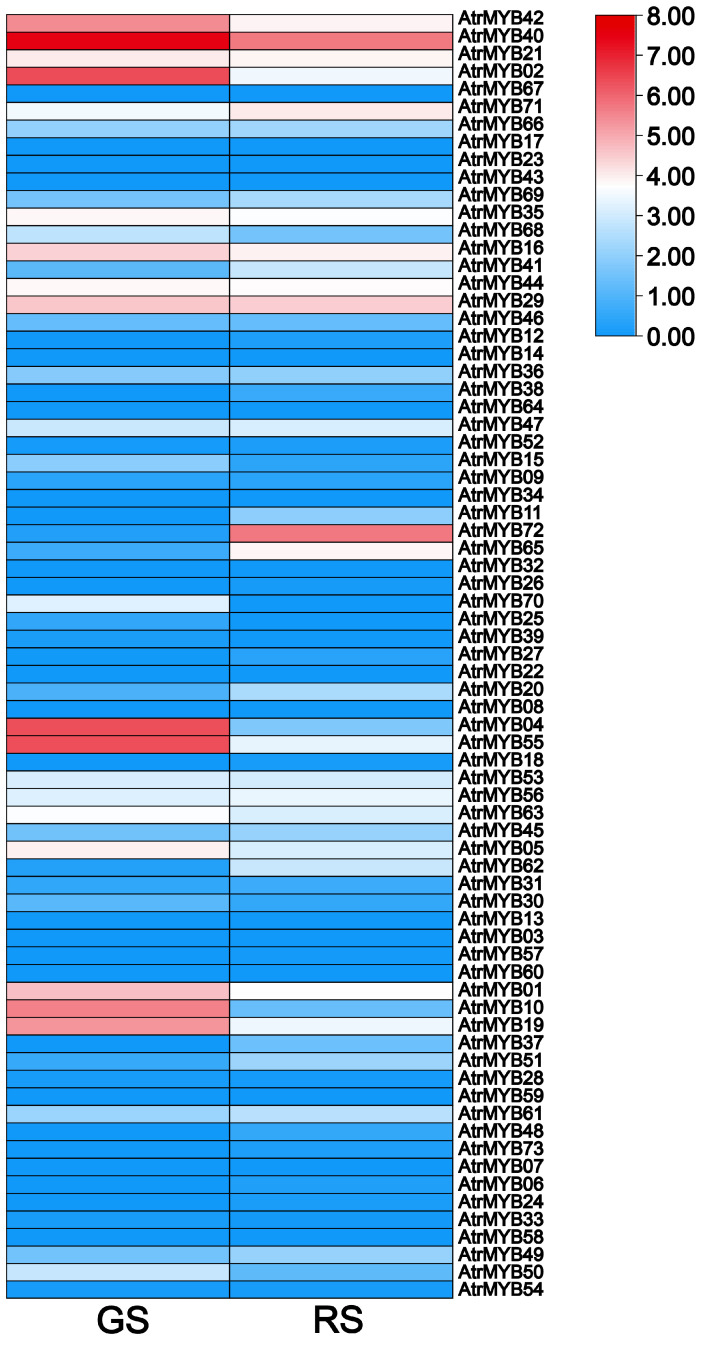
Expression patterns of the AtrMYBs.

An expression heat map of AtrMYB genes was generated based on the TPM values of the green section (GS) and the red section (RS) in the same leaf (Figure 7). They exhibited significantly different expression levels. In most cases, genes with the same subfamily displayed similar expression patterns. For example, the genes in subfamilies S2 (*AtrMYB04*, *AtrMYB55*), S4 (*AtrMYB01*, *AtrMYB10*, *AtrMYB19*, *AtrMYB37*) and S22 (*AtrMYB02*, *AtrMYB21*, *AtrMYB40*, *AtrMYB42*) exhibited a higher expression in the green section than in the red section in the same leaf. By contrast, the genes in subfamily S6 (*AtrMYB65*, *AtrMYB72*) showed a relatively higher expression in the red section than in the green section in the same leaf.

### 2.7. Gene Expression Validation (qRT-PCR)

**Figure 8:**
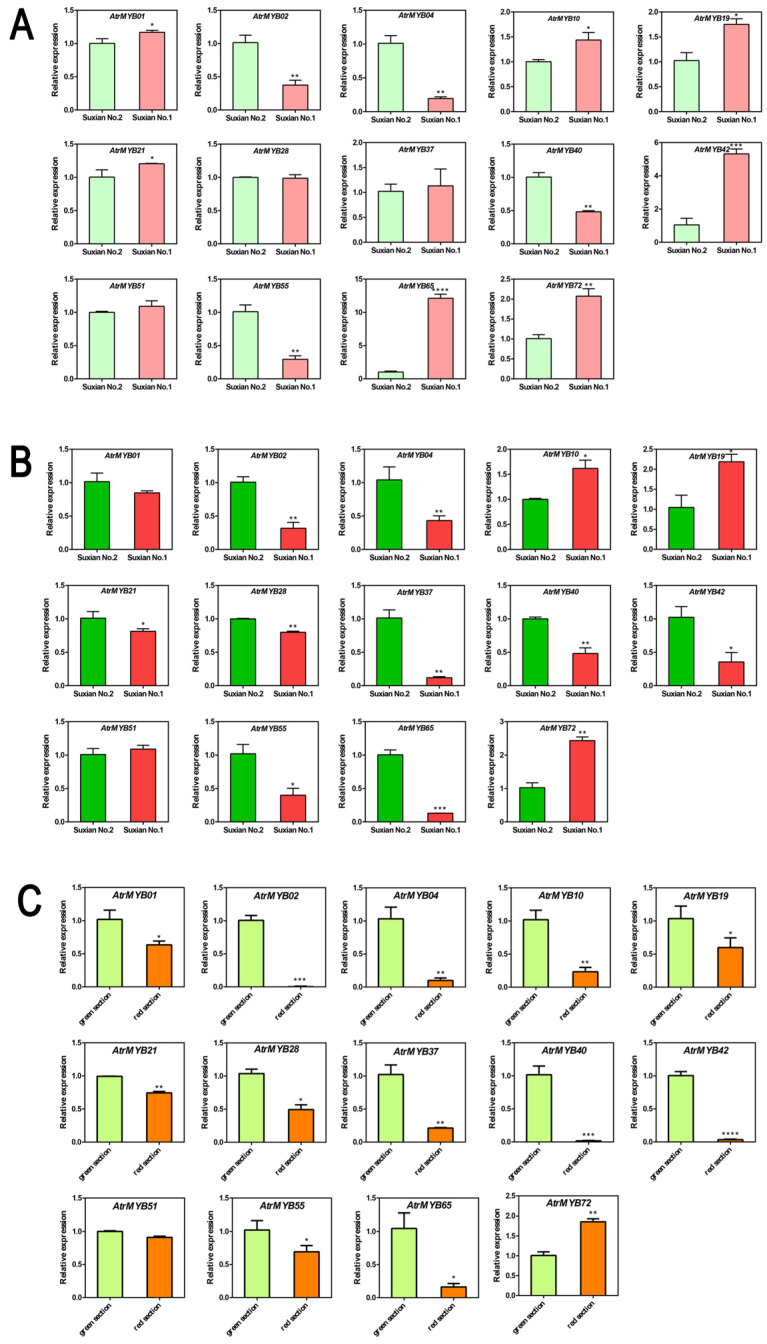
Quantitative analysis of selected *AtrR2R3-MYBs*. (**A**) Quantitative expression analysis in the leaves of ‘Suxian No.1’ and ‘Suxian No.2’; (**B**) Quantitative expression in the stems of ‘Suxian No.1’ and ‘Suxian No.2’; (**C**) Quantitative expression in the different sections of *Amaranthus* leaves. * indicates significant differences at *p* < 0.05, ** indicates significant differences at *p* < 0.01, *** indicates significant differences at *p* < 0.001, and **** indicates significant differences at *p* < 0.0001.

In order to analyze the expression levels of *R2R3-MYB* genes, qRT-PCR were performed to screen candidate *R2R3-MYB* genes involved in betalain biosynthesis of amaranth. As shown in Figure 8, the expression level of four *AtrMYBs* (*AtrMYB02*, *AtrMYB04*, *AtrMYB40* and *AtrMYB55*) was significantly lower in the leaf of ‘Suxian No.1’ than ‘Suxian No.2’, while the expression level of seven *AtrMYBs* (*AtrMYB01*, *AtrMYB10*, *AtrMYB19*, *AtrMYB21*, *AtrMYB42*, *AtrMYB65* and *AtrMYB72*) was significantly higher in the leaf of ‘Suxian No.1’ than ‘Suxian No.2’ (Figure 8A). The expression level of three *AtrMYBs* (*AtrMYB10*, *AtrMYB19* and *AtrMYB72*) was significantly lower in the stem of ‘Suxian No.1’ than ‘Suxian No.2’, while the expression level of ten *AtrMYBs* (*AtrMYB01*, *AtrMYB02*, *AtrMYB04*, *AtrMYB21*, *AtrMYB28*, *AtrMYB37*, *AtrMYB40*, *AtrMYB42*, *AtrMYB55* and *AtrMYB65*) was significantly lower in the leaf of ‘Suxian No.1’ than ‘Suxian No.2’ (Figure 8B). Only the expression level of *AtrMYB72* was significantly higher in the red section than the green section in the same leaf of amaranth. The other twelve *AtrMYBs* (*AtrMYB01*, *AtrMYB02*, *AtrMYB04*, *AtrMYB10*, *AtrMYB19*, *AtrMYB21*, *AtrMYB28*, *AtrMYB37*, *AtrMYB40*, *AtrMYB42*, *AtrMYB55* and *AtrMYB65*) were opposite (Figure 8C). These results suggested that most *AtrMYBs* might play a negative regulatory role in betalain biosynthesis. However, *AtrMYB72* was a positively regulatory transcript factor for the betalain biosynthesis in amaranth.

### 2.8. Functional Analysis of AtrMYB72 Gene

#### 2.8.1. Overexpression of *AtrMYB72* in Amaranth

**Figure 9:**
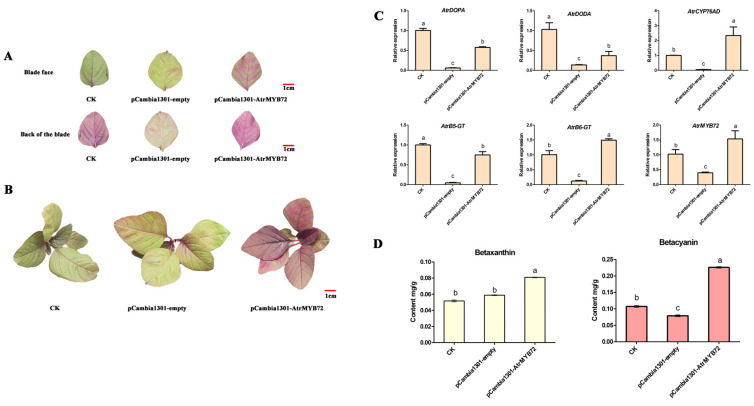
*Agrobacterium*-mediated transient transformation into the amaranth leaves revealing that the overexpression of *AtrMYB72* promotes the betalain synthesis in amaranth. (**A**) Plant leaves after transient transformation for 7 days. (**B**) Plants after transient transformation for 7 days. (**C**) Relative expression of betalain synthesis-related genes in leaves of plants with different transient transformations. (**D**) Betalain contents in the leaves with different transient transformation plants. (a, b and c indicate significant differences at *p* < 0.05; Bars: 1 cm).

Compared with the control and empty plasmid group, the leaves of overexpressing *AtrMYB72* plants were dark red (Figure 9A,B). Results of the qRT-PCR analyses showed that *AtrMYB72*, *AtrCYP76AD1* and *AtrB6-GT*, key genes involved in betalain metabolism, were upregulated in overexpressing *AtrMYB72* plants (Figure 9C). The betacyanin contents in transgenic plants were significantly higher (*p* < 0.05) than that in the control (Figure 9D).

#### 2.8.2. VIGS Analysis of *AtrMYB72*

**Figure 10:**
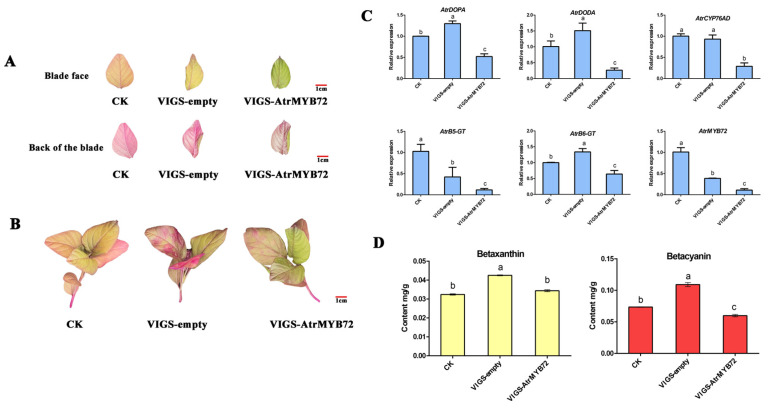
Silencing of *AtrMYB72* inhibited betalain synthesis. (**A**) control leaves (**left**), VIGS-empty leaves (**middle**) and VIGS-MYB72 leaves (**right**). (**B**) control plant (**left**), VIGS-empty plant (**middle**) and VIGS-MYB72 plant (**right**). (**C**) Relative expression levels of key genes involved in betalain synthesis in transgenic plants with gene silencing. (**D**) Betalain contents in leaves with gene silencing plants. Three biological replicates were performed for each sample (a, b and c indicate significant differences at *p* < 0.01; Bar = 1 cm).

The new leaves of ‘Suxian No.1’ were green after silencing of *AtrMYB72* for 2 weeks (Figure 10A,B). The qRT-PCR analysis showed that the expression levels of key genes in betalain synthesis were significantly decreased by gene silencing (Figure 10C). The betacyanin contents in silencing plants were significantly lower (*p* < 0.05) than that in the control (Figure 10D). These results indicate that *AtrMYB72* plays a key role in the betacyanin biosynthetic pathway of amaranth, and silencing *AtrMYB72* inhibited the betacyanin synthesis.

### 2.9. AtrMYB72 Binds to the Promoter Regions of the AtrCYP76AD1

**Figure 11:**
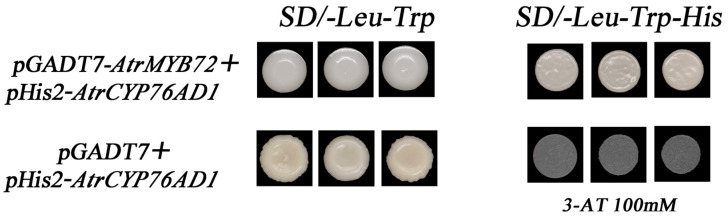
Y1H assay of *AtrMYB72* with *AtrCYP76AD1* promoter. The promoter of *AtrCYP76AD1* was constructed in the pHis2 vector, and the ORF of *AtrMYB72* was constructed in the pGADT7 vector. Yeast cells were cultured on an SD/-Leu-Trp-His medium supplemented with 100 nm of 3-AT.

A typical MBS motif was identified in the *AtrCYP76AD1* promoter, which is a cognate binding site for MYB TFs, suggesting that MYB TFs are involved in the regulation of *AtrCYP76AD1*. Therefore, the yeast one-hybrid (Y1H) assay was employed to further investigate the binding affinity of the *AtrMYB72* protein to *AtrCYP76AD1*. Firstly, we confirmed that 100 nm of 3-AT could inhibit the self-activation of pHis2-*AtrCYP76AD1*-Pro. The transformation yeast cells containing pHis2 *AtrCYP76AD1*-Pro + pGADT7-*AtrMYB72* were grown on an SD/-Leu-Trp-His (3-AT 100 nm) medium (Figure 11). The result verified that *AtrMYB72* was involved in the betalain biosynthesis in amaranth by binding directly the promoter region of *AtrCYP76AD1*.

### 2.10. AtrMYB72 Promoted the AtrCYP76AD1 Transcription

**Figure 12:**
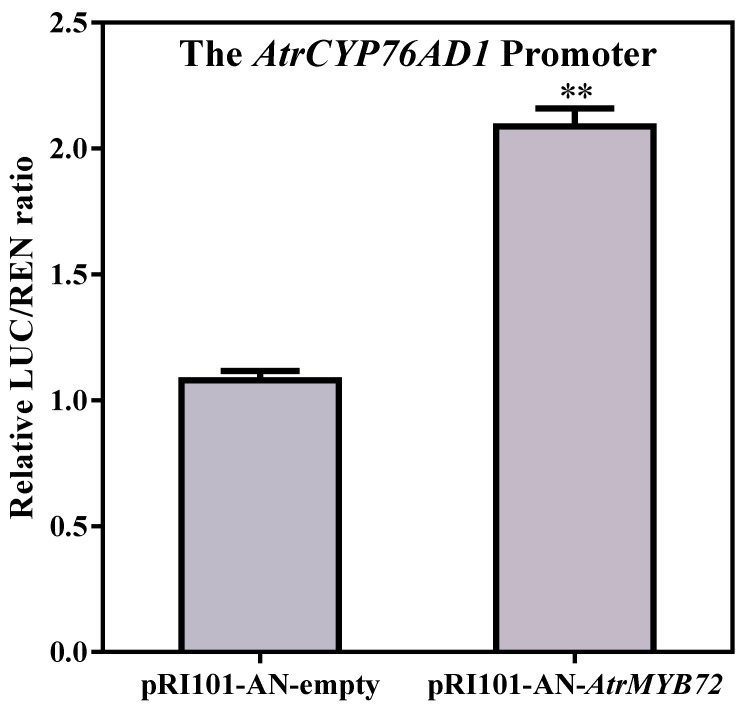
*AtrMYB72* promoted the *AtrCYP76AD1* transcription in *Nicotiana benthamiana* leaves. ** indicates significant differences at *p* < 0.01.

The transcriptional activation of *AtrMYB72* was confirmed in *Nicotiana benthamiana* leaves using the dual-luciferase reporter system (Figure 12). Compared with the negative control pRI 101-AN- empty, the transient expression of pRI 101-AN-*AtrMYB72* significantly promoted the value of the LUC/REN ratio driven by the promoter of *AtrCYP76AD1*. These results indicated that *AtrMYB72* participated in betalain biosynthesis by promoting the transcription of the *AtrCYP76AD1* gene.

## 3. Discussion

R2R3-MYB TF plays a crucial role in the growth and development, (a)biotic stress adaptation and secondary metabolism [48,49,50]. It supplies the most frequent members in the MYB transcription factor families in plants. With the completion of genome sequencing, the R2R3-MYB gene family has been identified and characterized in various plants, but has not been systematically studied in *Amaranthus tricolor*. In our study, a total of 73 R2R3-MYB members were identified. The number of gene members is similar to spinach (80) [51], quinoa (65) [52] and beet (70) [53], but less than *Arabidopsis thaliana* (126) [35] and pitaya (105) [44]. Due to the frequency of whole-genome duplication (WGD) events and lineage-specific expansion, the number of genes in the same gene family may vary significantly between plants. R2R3-MYBs in the amaranth genome may have been significantly influenced by the fragment replications; syntenic duplications mainly contribute to the expansion of *AtrMYB* genes. After gene duplication, the duplicates might undergo gene gain and loss events to result in phenotypic novelty within plants. Most AtrMYBs members were unstable, which were consistent with longan, poplar and cabbage [50,54,55]. Unstable proteins might be easier to modify to be involved in the regulation of the development and stress response [56]. Transcript factors, including R2R3-MYB, are predominantly localised in the nucleus. We predicted all AtrMYB proteins to be localised in the nucleus, which is consistent with previous studies in durian, pear and rice [57,58,59]. R2R3-MYB transcript factors were involved in growth and development, primary and secondary metabolisms and responses to (a)biotic stresses. Therefore, we deduced that AtrMYB proteins could participate in diverse plant biological functions.

We identified 73 R2R3-MYB members in *Amaranthus tricolor* through bioinformatics analysis, and characterized their phylogenetic relationships with *Arabidopsis thaliana*, pitaya and beet R2R3-MYBs. Our findings revealed that the R2R3-MYB domain of AtrMYBs exhibits a high degree of conservation with those of *Arabidopsis thaliana*, pitaya and beet R2R3-MYBs, especially pitaya and beet R2R3-MYBs. Nonetheless, we also observed divergence of R2R3-MYBs in *Amaranthus tricolor* compared with *Arabidopsis thaliana*, pitaya and beet, indicating a combination of conservation and diversity within plant R2R3-MYBs. Based on phylogenetic analysis, R2R3-MYB transcription factors were divided into 18 subgroups in *Amaranthus tricolor*, excluding S8, S10, S17, S3, S12, S15 and S16 in *Amaranthus tricolor*, which is consistent with pitaya and beet [44,53]. The same phylogenetic group possessed similar motif compositions, gene structures and gene functions, while the diversity in motif compositions and gene structures was observed between the R2R3-MYB family members in different phylogenetic groups [60]. In *Arabidopsis thaliana*, *AtMYB3*, *AtMYB4* [61], *AtMYB7* and *AtMYB32*, clustered in S4, belong to transcriptional repressors. *AtrMYB01*, *AtrMYB10*, *AtrMYB19* and *AtrMYB37* also clustered into S4 with the pitaya *HuMYB1*. It has been reported that *HuMYB1* could reduce the transcriptional activity of *HuADH1*, *HuCYP76AD1-1* and *HuDODA1* to inhibit the betalain biosynthesis in the ripening of pitaya pulp [44]. We detected the expression level of these genes in S4 showing that it was significantly lower in the red section than in the green section in *Amaranthus*. Furthermore, in *Arabidopsis thaliana*, *AtMYB75*/PAP1 [62], *AtMYB90*/PAP2 [63], *AtMYB113* and *AtMYB114* (S6) regulate anthocyanin biosynthesis [64]. *AtrMYB65* and *AtrMYB72* clustered into S6 with *BvMYB1* (Bv_ralf and Bv_jkkr), too. *BvMYB1* promoted betalain biosynthesis in beet. Our results showed *AtrMYB72* was involved in the betalain biosynthesis in amaranth by the expression analysis. Based on the comparison of DNA-binding domains, phylogenetic relationship and collinearity analysis between amaranth, pitaya, beet and *Arabidopsis thaliana*, the evolutionary relationship was closer between betalain-producing plants of amaranth, pitaya and beet compared to amaranth and the anthocyanin-producing plant *Arabidopsis thaliana* [35,44,53]. We supposed that evolutionary pressures played a vital role. These results support the deduction that the R2R3-MYB family underwent functional conservation and diversification during evolution.

**Figure 13:**
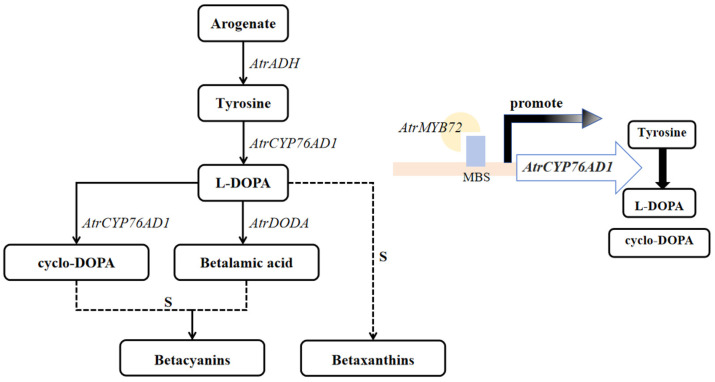
A hypothetical model of *AtrMYB72* gene regulated *AtrCYP76AD1* involved in betalain biosynthesis in amaranth. *AtrMYB72* transcript factor activated *AtrCYP76AD1* transcription by binding the MBS elements of the *AtrCYP76AD1* promoter.

According to the qRT-PCR results, the expression of the *AtrMYB72* gene was significantly higher in the leaves and stems of ‘Suxian No.1’ than of ‘Suxian No.2’, and the green section was significantly lower than that of the red section in *Amaranthus*. It is therefore deduced that the *AtrMYB72* transcription factor positively regulates the betalain biosynthesis in amaranth. We found that *AtrMYB72* overexpression could promote the betalain synthesis and increase the betalain content in ‘Suxian No.1’ amaranth. While the MYB domain of *AtrMYB72* was silenced by VIGS technology, betacyanin synthesis was inhibited, and the expression level of the key genes in the betalain synthesis pathway was down-regulated. Previous studies have shown that CYP76AD1 is the first step of the betacyanin synthesis, which catalyzes the L-tyrosine to L-DOPA [21]. And the promoter sequence of *AtrCYP76AD1* contains an MYB binding site (MBS) element. A yeast one-hybrid assay showed that *AtrMYB72* interacts with the *AtrCYP76AD1* promoter, providing evidence that *AtrMYB72* was involved in betalain biosynthesis in amaranth by activating the *AtrCYP76AD1* transcription. *AtrMYB72* transcript factor regulated *AtrCYP76AD1* transcription by binding the MBS elements of the *AtrCYP76AD1* promoter. The result of the dual luciferase assay showed that *AtrMYB72* promoted the transcription of *AtrCYP76AD1* (Figure 13). However, whether *AtrMYB72* activates other structural genes or *AtrMYB72* can coordinate with the other transcript factors to regulate betalain biosynthesis needs to be further investigated.

## 4. Materials and Methods

### 4.1. Material and Treatment

**Figure 14:**
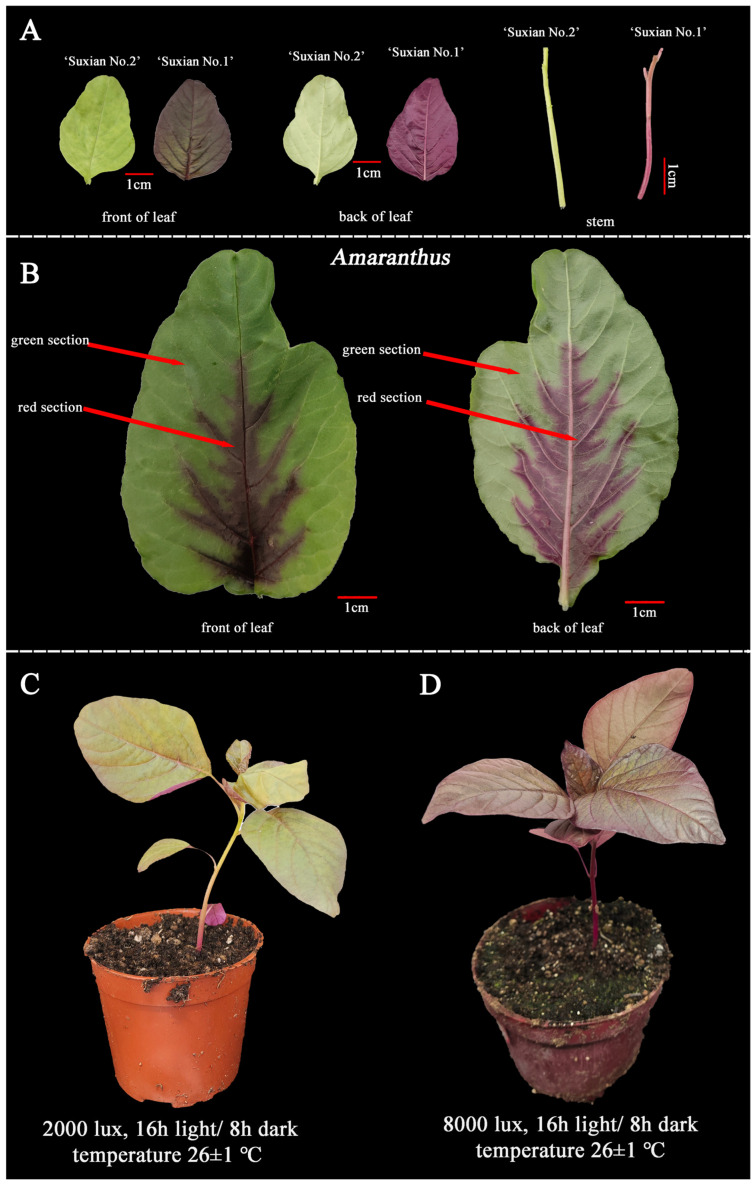
Plant phenotype of amaranth. (**A**) represents ‘Suxian No.1’ and ‘Suxian No.2’. (**B**) represents different parts in full-red amaranth leaves. (**C**) represents ‘Suxian No.1’ in (2000 lux, 16 h light/8 h dark, temperature 26 ± 1 °C). (**D**) represents ‘Suxian No.1’ in (8000 lux, 16 h light/8 h dark, temperature 26 ± 1 °C).

‘Suxian No.1’ and ‘Suxian No.2’ were materials which were provided by the Suzhou Academy of Agricultural Sciences. The ‘Suxian No.1’ is red and rich in betalain, and ‘Suxian No.2’ is green without betalain (Figure 14A). The red section of full-red amaranth leaf is rich in betalain, and the green section contains little or even no betalain in *Amaranthus* (Figure 14B). ‘Suxian No.1’ cultured in environment (2000 lux, 16 h light/8 h dark, temperature 26 ± 1 °C) was performed for transient transformation, and ‘Suxian No.1’ cultured in environment (8000 lux, 16 h light/8 h dark, temperature 26 ± 1 °C) was performed for virus-induced gene silencing (VIGS) (Figure 14C,D). All samples were frozen in liquid nitrogen immediately and stored at −80 °C for qRT-PCR analysis.

### 4.2. Identification of R2R3-MYB Gene Family Members in Amaranth

#### 4.2.1. Data Sources, Gene Identification and Physicochemical Properties Analysis

The genome database of *Amaranthus tricolor* was obtained from the AGIS website (ftp://ftp.agis.org.cn/~fanwei/Amaranthus_tricolor) (accessed on 21 June 2023) [65]. The plot hidden Markov model (HMM) profile of MYB DNA-binding domain (PF00249) was downloaded from Pfam (https://pfam.xfam.org/) (accessed on 27 September 2022) to identify MYB genes from the amaranth genome, with the critical E value set to <10^−5^ [49,66,67]. To ensure the presence of the core MYB domains, the putative MYB sequences were further screened for R2R3-MYB using SMART (https://smart.embl-heidelberg.de/) (accessed on 29 August 2023) and NCBI Conserved Structural Domain Database (NCBI-CDD, https://www.ncbi.nlm.nih.gov/cdd/) (accessed on 29 August 2023), and the sequences lacking MYB DNA-binding domain identified in the previous step were excluded [68]. The sequences with two MYB domains were considered as R2R3-MYB gene family members.

Physical parameters, including the molecular mass and theoretical isoelectric point (pI) of the deduced R2R3-MYB proteins were investigated using ExPASy (http://web.expasy.org/protparam/) (accessed on 4 September 2023).

#### 4.2.2. Chromosomal Locations and Gene Synteny Analyses

The chromosomal location of *R2R3-MYB* genes was identified on the amaranth genome and visualized with TBtools-II software v2.142 [69]. Duplicated gene pairs among the selected plants were identified using the MCScanX. The synteny analysis of the *R2R3-MYB* genes between amaranth and other species (*Arabidopsis thaliana*, *Beta vulgaris* and *Hylocereus undatus*) was visualized using TBtools-II software.

#### 4.2.3. Analysis of Conserved Motifs and Conserved Domains of R2R3-MYB Proteins

Conserved motifs of AtrMYB proteins were identified using MEME (Multiple Expectation Maximization for Motif Elicitation) [http://meme-suite.org/tools/meme] (accessed on 16 November 2024) and visualized using TBtools-II software. The DNA-binding domains of R2R3-MYB proteins were aligned by Cluster X 1.83 software and shown by WEBLOGO online tool (https://weblogo.berkeley.edu/logo.cgi) (accessed on 18 November 2024).

#### 4.2.4. Phylogenetic Analysis of AtrMYBs

A phylogenetic tree was constructed for the amino acid sequences of AtrMYBs from amaranth, *Arabidopsis thaliana*, beet and pitaya using the Maximum likelihood method (ML) in MEGA-X 10.2.6 software with 1000 bootstrap replications.

#### 4.2.5. Prediction of Cis-Regulatory Elements in Promoter Sequences of AtrMYBs

The 2 kb promotor region of each R2R3-MYB gene was submitted to the PlantCARE (http://bioinformatics.psb.ugent.be/webtools/plantcare/html/) (accessed on 10 September 2023) promoter analysis tool to identify potential *cis*-regulatory elements.

#### 4.2.6. Analysis of Expression Patterns of R2R3-MYB Gene Family

In order to investigate betalain biosynthesis, we evaluated published amaranth transcriptome data from the NCBI database (SRA: SRR924089-SSR924092) [70]. We reanalyzed this data in combination with amaranth genomic data, and obtained the TPM value to identify differentially expressed genes. A heatmap of their expression conditions in amaranth was again visualized using TBtools-II software.

#### 4.2.7. qRT-PCR Analyses

Total RNA was isolated from samples using MolPure Plant Plus RNA Kit (Yeasen, Shanghai, China) according to the manufacturer’s instructions. First-strand cDNA was synthesized from 1 mg of total RNA using Recombinant M-MLV reverse transcriptase Kit (TransGen Biotech, Beijing, China). Quantitative real time-PCR (qRT-PCR) was performed in optical 96-well plates using the Roche LightCycler 480 instrument (Roche, Solna, Sweden). The reactions were carried out in a 20 μL volume containing 10 μL of SYBR Premix Ex Taq, 0.8 μL of gene specific primers, 2 μL diluted cDNA and 6.4 μL of ddH_2_O. The qRT-PCR reaction procedure was as follows: pre-denaturation at 95 °C for 30 s, 45 cycles of denaturation at 95 °C for 10 s and annealing/extension at 58 °C for 20 s, followed at 72 °C for 12 s. Three biological repeats were performed for each treatment.

SAND was used as the internal reference gene [71]. The 2^−ΔΔCT^ method was used for the quantitative analyses of gene expression. The primer pairs used for the qRT-PCR analysis of *R2R3-MYB* genes are listed in Supplementary Table S1.

### 4.3. Functional Analysis of AtrMYB72

The primer pairs used for the vector construction are listed in Supplementary Table S2. pCambia1301-*AtrMYB72*-GUS and pCambia1301-GUS were transformed into the *Agrobacterium tumefaciens* strain GV3101, respectively. The bacterial cells were resuspended to an OD600 of 0.8–1.0 using MAA buffer (3% sugar, MS culture medium, 10 mM magnesium chloride, 200 mM acetylsyringone). Subsequently, bacterial cells were infiltrated into the amaranth leaves. Then, these seedlings were cultured in the dark at 25 °C for 1–2 days. These seedlings were then transferred to a culture room with 16 h/8 h (light/dark) at 25 °C. Plant phenotypes were observed. When the leaf color of the transiently transformed plants changed, the leaves were collected to detect gene expression levels and to determine the betalain content.

The gene fragment with a conserved domain of *AtrMYB72* was ligated to the pTRV2 vector. pTRV1, pTRV2 and pTRV2-*AtrMYB72* were transformed into *Agrobacterium tumefaciens* strain GV3101. Bacterial cells were resuspended to an OD600 of 0.8–1.0 using the MAA buffer. pTRV2 (negative control) and pTRV2- *AtrMYB72* were separately infiltrated into amaranth leaves with pTRV1 in a ratio of 1:1. The plants were cultured in the dark for 2 days; subsequently, they were transferred into culture chambers (16 h light/8 h dark) for 2 weeks to observe plant phenotypes. When the leaf color changed after injection, the leaves were collected to detect the gene expression levels and determine the betalain content.

### 4.4. Yeast One-Hybrid Assay

Yeast one-hybrid analysis was performed using an Y187- pHis2 Yeast One-Hybrid interaction proving kit (Coolaber, Beijing, China). The promoter of *AtrCYP76AD1* was inserted into the pHis2 vector. Then, the recombinant pHis2-*AtrCYP76AD1* plasmid was transformed into the yeast strain Y187 to obtain the bait reporter strain (for primers, see Supplementary Table S3). The minimum inhibitory concentration (0–150 nm) of 3-amino-1,2,4-triazole (3-AT) was measured on an SD/-Trp-His medium. The coding regions of *AtrMYB72* was cloned into the pGADT7 vector as prey plasmids and the pGADT7-*AtrMYB72* vector plasmid was used to transform the positively verified *AtrCYP76AD1*- pHis2. The bait yeast strain Y187 was cultured on SD/-Leu-Trp-His medium plates containing 100 nm 3-AT for 3–5 days at 30 °C.

### 4.5. Dual-Luciferase Transient Expression Assay

The full-length coding sequence of *AtrMYB72*, as effector, was cloned with pRI101-AN. And the *AtrCYP76AD1* promoter, as reporter, was cloned within pGreenII 0800-LUC vector (primers are listed in Supplementary Table S4). The recombinant plasmid was transferred into *Agrobacterium* GV3103. The effector and reporter were infiltrated into *Nicotiana benthamiana* leaves with a mixture of 3:1, and LUC and REN activities were measured by a Dual Luciferase reporter gene assay kit (Yeasen, Shanghai, China) after 48 h. Three independent experiments were carried out with at least three biological replicates per experiment.

### 4.6. Statistical Analysis

Data were analyzed using IBM SPSS Statistics 26 software. One-way ANOVAs was utilized to evaluate the differences, it was significant at *p* ≤ 0.05. GraphPad Prism 6.01 was used to generate histograms.

## 5. Conclusions

A total of 73 *R2R3-MYB* genes were obtained based on the amaranth genome, distributed on 17 chromosomes of amaranth, all with highly conserved R2 and R3 repeats. These R2R3-MYB were located in the nucleus. According to AtrMYB function, they were divided into 7 groups and 18 subgroups, consisting of conserved motif. Thirteen genes (*AtrMYB01*, *AtrMYB02*, *AtrMYB04*, *AtrMYB10*, *AtrMYB19*, *AtrMYB21*, *AtrMYB28*, *AtrMYB37*, *AtrMYB40*, *AtrMYB42*, *AtrMYB55*, *AtrMYB65*, *AtrMYB72*) were further analyzed by qRT-PCR. The expression level of *AtrMYB72* in ‘Suxian No.1’ was significantly higher than that in ‘Suxian No.2’. *AtrMYB72* promoted betalain biosynthesis by binding the MBS elements of the *AtrCYP76AD1* promoter to activate the *AtrCYP76AD1* transcription in amaranth. In conclusion, this study is the first report on the genome-wide analysis of the *R2R3-MYB* gene family, and it can provide valuable information for a better understanding of the MYB transcript factor involved in betalain biosynthesis in amaranth.

## Data Availability

All datasets generated for this study are included in the article.

## Supplementary Materials

The following supporting information can be downloaded at https://www.mdpi.com/article/10.3390/plants14030324/s1, Supplementary Table S1: Primer pairs for gene expression; Supplementary Table S2: Primer pairs for vector construction; Supplementary Table S3: Primer pairs for yeast one hybrid; Supplementary Table S4: Primer pairs for dual luciferase reporter gene assay; Supplementary Table S5: Physicochemical properties analysis of R2R3-MYB in *Amaranthus tricolor* L.

## References

[1] Parveen M., Ray S., Chatterjee N.C. (2018). Detection and diversity pattern of amaranth Cultivars originated in diverse region of Indian subcontinent. Int. J. Pharma Bio Sci..

[2] Nazeer S., Zubair Akram M., Ali M. (2022). Amaranth as Nutrition-Rich and Climatic Resilient Crop: A Review. ACS Agric. Conspec. Sci..

[3] Liu S., Wang X., Peng L. (2023). Comparative Transcriptomic Analysis of the Metabolism of Betalains and Flavonoids in Red Amaranth Hypocotyl under Blue Light and Dark Conditions. Molecules.

[4] Silva A.D., Ávila S., Küster R.T., dos Santos M.P., Grassi M.T., Pinto C.d.Q.P., Miguel O.G., Ferreira S.M.R. (2021). In vitro Bioaccessibility of Proteins, Phenolics, Flavonoids and Antioxidant Activity of Amaranthus viridis. Plant Food Hum. Nutr..

[5] Xuan Y., Feng W., Lai Z., Liu S. (2024). Effects of aromatic amino acids on callus growth and accumulation of secondary metabolites in amaranth. Trop. Plants.

[6] Xuan Y., Liu S., Xie L., Pan J. (2023). Establishment of *Amaranthus* spp. calluses and cell suspension culture, and the effect of plant growth regulators on total flavonoid content. Trop. Plants.

[7] Polturak G., Aharoni A. (2019). Advances and future directions in betalain metabolic engineering. New Phytol..

[8] Adhikary D., Khatri-Chhetri U., Tymm F.J.M., Murch S.J., Deyholos M.K. (2019). A virus-induced gene-silencing system for functional genetics in a betalainic species, *Amaranthus tricolor* (Amaranthaceae). Appl. Plant Sci..

[9] Murthy H.N., Joseph K.S., Paek K.Y., Park S.-Y. (2024). Correction to: Production of betalains in plant cell and organ cultures: A review. Plant Cell Tissue Organ Cult..

[10] Gómez-Maqueo A., Welti-Chanes J., Cano M.P. (2020). Release mechanisms of bioactive compounds in fruits submitted to high hydrostatic pressure: A dynamic microstructural analysis based on prickly pear cells. Food Res. Int..

[11] Babaei M., Thomsen P.T., Dyekjær J.D., Glitz C.U., Pastor M.C., Gockel P., Körner J.D., Rago D., Borodina I. (2023). Combinatorial engineering of betalain biosynthesis pathway in yeast Saccharomyces cerevisiae. Biotechnol. Biofuels Bioprod..

[12] Ponce-Martínez A.J., Rodríguez-Párraga J., Solivella-Poveda A.M., Fernández-López J.A., -Martos M.V., Pérez-Alvarez J.A. (2023). Beetroot juices as colorant in plant-based minced meat analogues: Color, betalain composition and antioxidant activity as affected by juice type. Food Biosci..

[13] Imamura T., Isozumi N., Higashimura Y., Koga H., Segawa T., Desaka N., Takagi H., Matsumoto K., Ohki S., Mori M. (2022). Red-Beet Betalain Pigments Inhibit Amyloid-β Aggregation and Toxicity in Amyloid-β Expressing Caenorhabditis elegans. Plant Food Hum. Nutr..

[14] Gao Y., Liang X., Tian Z., Ma Y., Sun C. (2021). Betalain exerts cardioprotective and anti-inflammatory effects against the experimental model of heart failure. Hum. Exp. Toxicol..

[15] Thiruvengadam M., Chung I.-M., Samynathan R., Chandar S.R.H., Venkidasamy B., Sarkar T., Rebezov M., Gorelik O., Shariati M.A., Simal-Gandara J. (2024). A comprehensive review of beetroot (Beta vulgaris L.) bioactive components in the food and pharmaceutical industries. Crit. Rev. Food Sci..

[16] Li G., Meng X., Zhu M., Li Z. (2019). Research Progress of Betalain in Response to Adverse Stresses and Evolutionary Relationship Compared with Anthocyanin. Molecules.

[17] Gliszczyńska-Šwigło A., Szymusiak H., Malinowska P. (2006). Betanin, the main pigment of red beet: Molecular origin of its exceptionally high free radical-scavenging activity. Food Addit. Contam..

[18] Lopez-Nieves S., Yang Y., Timoneda A., Wang M., Feng T., Smith S.A., Brockington S.F., Maeda H.A. (2018). Relaxation of tyrosine pathway regulation underlies the evolution of betalain pigmentation in Caryophyllales. New Phytol..

[19] Teng X.-L., Chen N., Xiao X.-G. (2016). Identification of a Catalase-Phenol Oxidase in Betalain Biosynthesis in Red Amaranth (Amaranthus cruentus). Front. Plant Sci..

[20] Polturak G., Breitel D., Grossman N., Sarrion-Perdigones A., Weithorn E., Pliner M., Orzaez D., Granell A., Rogachev I., Aharoni A. (2016). Elucidation of the first committed step in betalain biosynthesis enables the heterologous engineering of betalain pigments in plants. New Phytol..

[21] Sunnadeniya R., Bean A., Brown M., Akhavan N., Hatlestad G., Gonzalez A., Symonds V.V., Lloyd A. (2016). Tyrosine Hydroxylation in Betalain Pigment Biosynthesis Is Performed by Cytochrome P450 Enzymes in Beets (Beta vulgaris). PLoS ONE.

[22] Brockington S.F., Yang Y., Gandia-Herrero F., Covshoff S., Hibberd J.M., Sage R.F., Wong G.K.S., Moore M.J., Smith S.A. (2015). Lineage-specific gene radiations underlie the evolution of novel betalain pigmentation in Caryophyllales. New Phytol..

[23] Polturak G., Aharoni A. (2018). “La Vie en Rose”: Biosynthesis, Sources, and Applications of Betalain Pigments. Mol. Plant.

[24] Zheng X., Liu S., Cheng C., Guo R., Chen Y., Xie L., Mao Y., Lin Y., Zhang Z., Lai Z. (2016). Cloning and expression analysis of betalain biosynthesis genes in Amaranthus tricolor. Biotechnol. Lett..

[25] Chang Y.-C., Chiu Y.-C., Tsao N.-W., Chou Y.-L., Tan C.-M., Chiang Y.-H., Liao P.-C., Lee Y.-C., Hsieh L.-C., Wang S.-Y. (2021). Elucidation of the core betalain biosynthesis pathway in Amaranthus tricolor. Sci Rep-Uk.

[26] Tossi V.E., Tosar L.M., Pitta-Álvarez S.I., Causin H.F. (2021). Casting light on the pathway to betalain biosynthesis: A review. Environ. Exp. Bot..

[27] Xie F., Chen C., Chen J., Chen J., Hua Q., Shah K., Zhang Z., Zhao J., Hu G., Chen J. (2023). Betalain biosynthesis in red pulp pitaya is regulated via HuMYB132: A R-R type MYB transcription factor. BMC Plant Biol..

[28] Hatlestad G.J., A Akhavan N., Sunnadeniya R.M., Elam L., Cargile S., Hembd A., Gonzalez A., McGrath J.M., Lloyd A.M. (2015). The beet Y locus encodes an anthocyanin MYB-like protein that activates the betalain red pigment pathway. Nat. Genet..

[29] Chen C., Xie F., Shah K., Hua Q., Chen J., Zhang Z., Zhao J., Hu G., Qin Y. (2022). Genome-Wide Identification of WRKY Gene Family in Pitaya Reveals the Involvement of HmoWRKY42 in Betalain Biosynthesis. Int. J. Mol. Sci..

[30] Zhang L., Chen C., Xie F., Hua Q., Zhang Z., Zhang R., Chen J., Zhao J., Hu G., Qin Y. (2021). A Novel WRKY Transcription Factor HmoWRKY40 Associated with Betalain Biosynthesis in Pitaya (*Hylocereus monacanthus*) through Regulating HmoCYP76AD1. Int. J. Mol. Sci..

[31] Zeng J., Chen J., Shah K., Xie F., Chen C., Chen J., Zhao J., Hu G., Zhang Z., Qin Y. (2023). Identification ofHuSPL family and key role ofHuSPL12in regulation of betalain biosynthesis in pitaya. Physiol. Plant..

[32] Chen J., Xie F., Shah K., Chen C., Zeng J., Chen J., Zhang Z., Zhao J., Hu G., Qin Y. (2023). Identification of HubHLH family and key role of HubHLH159 in betalain biosynthesis by activating the transcription of HuADH1, HuCYP76AD1-1, and HuDODA1 in pitaya. Plant Sci..

[33] Yang R., Huang T., Song W., An Z., Lai Z., Liu S. (2023). Identification of WRKY gene family members in amaranth based on a transcriptome database and functional analysis of AtrWRKY42-2 in betalain metabolism. Front. Plant Sci..

[34] Dubos C., Stracke R., Grotewold E., Weisshaar B., Martin C., Lepiniec L. (2010). MYB transcription factors in Arabidopsis. Trends Plant Sci..

[35] Stracke R., Werber M., Weisshaar B. (2001). The R2R3-MYB gene family in Arabidopsis thaliana. Curr. Opin. Plant Biol..

[36] Yang J., Xu J., Zhang Y., Cui J., Hu H. (2022). Transcriptome-wide identification, characterization, and expression analysis of R2R3-MYB gene family during lignin biosynthesis in Chinese cedar (Cryptomeria fortunei Hooibrenk). Ind. Crop. Prod..

[37] Millard P.S., Kragelund B.B., Burow M. (2019). R2R3 MYB Transcription Factors–Functions outside the DNA-Binding Domain. Trends Plant Sci..

[38] Wu X., Xia M., Su P., Zhang Y., Tu L., Zhao H., Gao W., Huang L., Hu Y. (2024). MYB transcription factors in plants: A comprehensive review of their discovery, structure, classification, functional diversity and regulatory mechanism. Int. J. Biol. Macromol..

[39] Chen C., Zhang K., Khurshid M., Li J., He M., Georgiev M.I., Zhang X., Zhou M. (2019). MYB Transcription Repressors Regulate Plant Secondary Metabolism. Crit. Rev. Plant Sci..

[40] Hughes C.L., Harmer S.L. (2023). Myb-like transcription factors have epistatic effects on circadian clock function but additive effects on plant growth. Plant Direct.

[41] Biswas D., Gain H., Mandal A. (2023). MYB transcription factor: A new weapon for biotic stress tolerance in plants. Plant Stress.

[42] Huang X., Yang Q., Gao H. (2023). Research progress in the regulation of secondary metabolism in medicinal plants by MYB transcription factors. J. Holist. Integr. Pharm..

[43] Lloyd A., Brockman A., Aguirre L., Campbell A., Bean A., Cantero A., Gonzalez A. (2017). Advances in the MYB–bHLH–WD Repeat (MBW) Pigment Regulatory Model: Addition of a WRKY Factor and Co-option of an Anthocyanin MYB for Betalain Regulation. Plant Cell Physiol..

[44] Xie F., Hua Q., Chen C., Zhang Z., Zhang R., Zhao J., Hu G., Chen J., Qin Y. (2021). Genome-Wide Characterization of R2R3-MYB Transcription Factors in Pitaya Reveals a R2R3-MYB Repressor HuMYB1 Involved in Fruit Ripening through Regulation of Betalain Biosynthesis by Repressing Betalain Biosynthesis-Related Genes. Cells-Basel.

[45] Xie F., Shah K., Chen C., Sabir I.A., Chen J., Chen J., Chen J., Qin Y. (2024). Unraveling betalain suppression in pitaya: Insights from co-activatorHuMYB9 binding atHuCYP76AD1-1, HuADH1, andHuDODA1 super-enhancers. Food Qual. Saf..

[46] Xie L.L.S.B. (2016). Acta Botanica Boreali-Occidentalia Sin., D.S.; Z. Cloning and expression analysis of betalain-related transcription factor gene AmMYB1 in *Amaranthus tricolor* L.. Acta Bot. Boreali-Occident. Sin..

[47] Peng L.Y., Wang Y., Sun X.L., Xiao W., Zhao C.L., Wang C. (2019). Expression and Functional Analysis of AmMYB2 Related to Betalain Metabolism of *Amaranthus tricolor* L.. Acta Hortic. Sin..

[48] Zhao X., Wang S., Zhang H., Dong S., Chen J., Sun Y., Zhang Y., Liu Q. (2024). Genome-wide identification, expression analysis of the R2R3-MYB gene family and their potential roles under cold stress in Prunus sibirica. BMC Genom..

[49] Wang B., Xiong C., Peng Z., Luo Z., Wang X., Peng S., Yu Z. (2024). Genome-wide analysis of R2R3-MYB transcription factors in poplar and functional validation of PagMYB147 in defense against Melampsora magnusiana. Planta.

[50] Luo Y., Xu X., Yang L., Zhu X., Du Y., Fang Z. (2024). A R2R3-MYB transcription factor, FeR2R3-MYB, positively regulates anthocyanin biosynthesis and drought tolerance in common buckwheat (*Fagopyrum esculentum*). Plant Physiol. Biochem..

[51] Zhang Z., Liu Z., Wu H., Xu Z., Zhang H., Qian W., Gao W., She H. (2024). Genome-Wide Identification and Characterization of MYB Gene Family and Analysis of Its Sex-Biased Expression Pattern in *Spinacia oleracea* L.. Int. J. Mol. Sci..

[52] Liu Y., Wang M., Huang Y., Zhu P., Qian G., Zhang Y., Li L. (2023). Genome-Wide Identification and Analysis of R2R3-MYB Genes Response to Saline–Alkali Stress in Quinoa. Int. J. Mol. Sci..

[53] Stracke R., Holtgräwe D., Schneider J., Pucker B., Sörensen T.R., Weisshaar B. (2014). Genome-wide identification and characterisation of R2R3-MYB genes in sugar beet (*Beta vulgaris*). BMC Plant Biol..

[54] Lv X., Tian S., Huang S., Wei J., Han D., Li J., Guo D., Zhou Y. (2023). Genome-wide identification of the longan R2R3-MYB gene family and its role in primary and lateral root. BMC Plant Biol..

[55] Lei S., Li G., Jiang D., Yuan F., Zhou X., Zheng Y., Zhang H., Cao B. (2024). The Genome-Wide Identification of the R2R3-MYB Gene Family in Chinese Flowering Cabbage and the Characterization of Its Response to Pectobacterium carotovorum Infection. Horticulturae.

[56] Díaz V.M., Viñas-Castells R., García De Herreros A. (2014). Regulation of the protein stability of EMT transcription factors. Cell Adhes. Migr..

[57] Ali N.A.W.A., Wong G.R., Rahim A.N., Teoh S.H., Tan B.C., Lum W.S., Ho P.W.C., Mazumdar P. (2024). Genome-Wide Analysis of the R2R3-MYB Gene Family in Durian (Durio zibethinus) and Potential Role in Nutrient Stress Response. Trop. Plant Biol..

[58] Liu X., Huang Q., Liang Y., Lu Z., Liu W., Yuan H., Li H. (2024). Genome-Wide Identification and Expression Analysis of ‘NanGuo’ Pear Revealed Key MYB Transcription Factor Family Genes Involved in Anthocyanin Accumulation. Horticulturae.

[59] Zhang H.-C., Gong Y.-H., Tao T., Lu S., Zhou W.-Y., Xia H., Zhang X.-Y., Yang Q.-Q., Zhang M.-Q., Hong L.-M. (2024). Genome-wide identification of R2R3-MYB transcription factor subfamily genes involved in salt stress in rice (*Oryza sativa* L.). BMC Genom..

[60] Jintao F., Chenxi J., Jihong X., Jingao D. (2014). Structure and function of the 22nd subfamily in Arabidopsis R2R3-MYB family. Yíchuán.

[61] Chen Y., Chen Z., Kang J., Kang D., Gu H., Qin G. (2013). AtMYB14 Regulates Cold Tolerance in Arabidopsis. Plant Mol. Biol. Rep..

[62] Xing M., Xin P., Wang Y., Han C., Lei C., Huang W., Zhang Y., Zhang X., Cheng K., Zhang X. (2024). A negative feedback regulatory module comprising R3-MYB repressor MYBL2 and R2R3-MYB activator PAP1 fine-tunes high light-induced anthocyanin biosynthesis in Arabidopsis. J. Exp. Bot..

[63] Velten J., Cakir C., Cazzonelli C.I. (2010). A Spontaneous Dominant-Negative Mutation within a 35S::AtMYB90 Transgene Inhibits Flower Pigment Production in Tobacco. PLoS ONE.

[64] Muñoz-Gómez S., Suárez-Baron H., Alzate J.F., González F., Pabón-Mora N. (2021). Evolution of the Subgroup 6 R2R3-MYB Genes and Their Contribution to Floral Color in the Perianth-Bearing Piperales. Front. Plant Sci..

[65] Wang H., Xu D., Wang S., Wang A., Lei L., Jiang F., Yang B., Yuan L., Chen R., Zhang Y. (2023). Chromosome-scale *Amaranthus tricolor* genome provides insights into the evolution of the genus Amaranthus and the mechanism of betalain biosynthesis. DNA Res..

[66] Mistry J., Chuguransky S., Williams L., Qureshi M., Salazar G.A., Sonnhammer E.L.L., Tosatto S.C., Paladin L., Raj S., Richardson L.J. (2021). Pfam: The protein families database in 2021. Nucleic Acids Res..

[67] Xie L., Wang Y., Tao Y., Chen L., Lin H., Qi Z., Li J. (2024). Genome-wide identification and analysis of anthocyanin synthesis-related R2R3-MYB genes in Fragaria pentaphylla. BMC Genom..

[68] Li X.J., Zhou X.H., Bao A.K. (2024). Genome-wide analysis of the R2R3-MYB gene family and identification of candidate genes that regulate isoflavone biosynthesis in red clover (*Trifolium pratense*). Int. J. Biol. Macromol..

[69] Chen C., Wu Y., Li J., Wang X., Zeng Z., Xu J., Liu Y., Feng J., Chen H., He Y. (2023). TBtools-II: A “one for all, all for one” bioinformatics platform for biological big-data mining. Mol. Plant.

[70] Liu S., Zheng X., Pan J., Peng L., Cheng C., Wang X., Zhao C., Zhang Z., Lin Y., XuHan X. (2019). RNA-sequencing analysis reveals betalains metabolism in the leaf of *Amaranthus tricolor* L.. PLoS ONE.

[71] Xiao F., Zheng Y., Chen J., Zhao C., Chen H., Wang L., Liu S. (2021). Selection and validation of reference genes in all-red Amaranth (*Amaranthus tricolor* L.) seedlings under different culture conditions. J. Hortic. Sci. Biotechnol..

